# B Cells as Prognostic Biomarker After Surgery for Colorectal Liver Metastases

**DOI:** 10.3389/fonc.2020.00249

**Published:** 2020-03-05

**Authors:** Joost Hof, Lydia Visser, Diederik J. Höppener, Pieter M. H. Nierop, Miente M. Terpstra, Annette S. H. Gouw, Dirk J. Grünhagen, Cornelis Verhoef, Rolf H. Sijmons, Koert P. de Jong, Klaas Kok

**Affiliations:** ^1^Department of Genetics, University Medical Center Groningen, University of Groningen, Groningen, Netherlands; ^2^Department of Hepato-Pancreato-Biliary Surgery and Liver Transplantation, University Medical Center Groningen, University of Groningen, Groningen, Netherlands; ^3^Department of Pathology and Medical Biology, University Medical Center Groningen, University of Groningen, Groningen, Netherlands; ^4^Department of Surgical Oncology, Erasmus MC Cancer Institute, Rotterdam, Netherlands

**Keywords:** colorectal liver metastases, cancer, genetics, RNA sequencing, B cells, immunohistochemistry

## Abstract

**Background:** The aim of this study was to identify more accurate variables to improve prognostication of individual patients with colorectal liver metastases (CRLM). Clinicopathological characteristics only partly explain the large range in survival rates.

**Methods:** MessengerRNA expression profiles of resected CRLM of two patient groups were analysed by mRNA sequencing: poor survivors (death from recurrent disease <30 months after surgery) and good survivors (no recurrent disease >60 months after surgery). Tumour and adjacent liver parenchyma samples were analysed.

**Results:** MessengerRNA expression profiling of the tumour samples identified 77 genes that were differentially expressed between the two survival groups at a False Discovery Rate (FDR) <0.1. In the adjacent liver parenchyma samples only one gene, *MTRNR2L1*, showed significantly higher expression in the good survivors. Pathway analysis showed higher expression of immune-related and stroma-related genes in tumour samples from good survivors. Expression data was then validated by immunohistochemistry in two cohorts comprising a total of 125 patients. Immunohistochemical markers that showed to be associated with good survival in the total cohort were: high K/L+ infiltration in tumour stroma [*p* = 0.029; OR 2.500 (95% CI 1.100–5.682)] and high CD79A+ infiltration in tumour stroma [*p* = 0.036; OR 2.428 (95%CI 1.062–5.552)].

**Conclusions:** A high stromal infiltration of CD79A+ B cells and K/L+ plasma cells might be favourable prognostic biomarkers after surgery for CRLM.

## Introduction

Colorectal cancer is the cancer with the third highest incidence in Europe, and it often disseminates to the liver (1). Curative treatment by liver surgery is possible in about 10–20% of patients with colorectal liver metastases (CRLM) (2, 3) and survival rates after surgery have improved in the last decades, with reported 5-year survival rates ranging from 14 to 60% (4–6). Clinicopathological characteristics can partly explain this large range, but they are inconsistent in accurately determining the prognosis for the individual patient (7).

Recent advances in technology have led to the notion that molecular characteristics might outperform clinicopathological scoring systems in predicting patient treatment and survival (8). Four individual centres have published prognostic gene signatures for colorectal liver metastases based on expression microarrays, but no single gene was shared between the four signatures (9–12).

As early as 1889, Paget suggested that it is not only the metastatic tumour (the “seed”) but also the hosting organ or tissue (the “soil”) that might be important for metastatic tumour growth (13). Progression of remaining, clinically undetectable metastatic tumour cells in the remnant liver after liver surgery is dependent on both micrometastases and on a favourable micro-environment (14–16). For example, it has been shown that microRNA expression in adjacent liver parenchyma can be associated with recurrent disease and patient survival (17).

In this study, we applied genome-wide mRNA expression profiling by RNAseq in two patient groups selected on survival after liver surgery for CRLM: poor survivors, those who died from recurrences within 30 months after surgery, and good survivors, those alive without recurrences 60 months or more after surgery. Samples from both tumour tissue and adjacent liver parenchyma were included. Immunohistochemistry was performed in two cohorts to validate the RNAseq results on protein level. The goal of this study was to identify molecular markers for favourable patient survival.

## Materials and Methods

### Patients and Samples

In this study we included patients from two tertiary referral centres for liver surgery, the University Medical Centre Groningen (UMCG, cohort 1) and the Erasmus Medical Centre in Rotterdam (cohort 2). Patients were selected from prospectively maintained databases. Inclusion criteria were (1) R0 partial liver resection for CRLM, (2) no neoadjuvant chemotherapy before liver surgery and no adjuvant chemotherapy after liver surgery, (3) a Fong clinical risk score (5) of 3 or lower, (4) no detectable extrahepatic disease at time of surgery, (5) no other known malignant disease, and (6) availability of fresh frozen (−80°C) resected CRLM material. Of note, (neo)adjuvant chemotherapy is not a standard treatment in the Netherlands. None of the patients received perioperative chemotherapy; neither neoadjuvant nor adjuvant after liver surgery. Adjuvant chemotherapy after primary tumor resection was allowed provided that it was not within 6 months before liver surgery. Follow-up consisted of cross-sectional imaging and measurement of carcinoembryonic antigen (CEA) serum levels every 3–4 months during the first 2 years after liver resection and at 6-month intervals afterwards up to 5 years. Samples of two groups of patients with different survival rates were selected: poor survivors, who died of recurrent disease within 30 months after partial liver resection, and good survivors, who showed no evidence of recurrent disease at 60 months after liver resection. The samples of tumour tissue and adjacent liver parenchyma were reviewed by an experienced hepatopathologist to judge the quality of the tissue. Genome-wide mRNA expression profiling by RNAseq followed by immunohistochemistry was performed on a cohort of patients treated in the University Medical Centre Groningen (cohort 1). Corroboration of immunohistochemical results was performed using tissue samples from patients with similar inclusion criteria who underwent liver surgery in another tertiary referral centre for liver surgery in the Netherlands (cohort 2, Erasmus Medical Centre in Rotterdam). Baseline clinicopathological characteristics of both cohorts are summarised in Table 1.

**Table 1:** Cohorts 1 and 2.

	**Cohort 1 (*n* = 47)**	**Cohort 2 (*n* = 78)**	***P***
**PATIENT CHARACTERISTICS**			
Mean age at time of liver surgery	62.5 ± 9.7	67.7 ± 9.9	0.005
Male sex	23 (48.9%)	50 (64.1%)	0.096
**TUMOUR CHARACTERISTICS**			
Major liver surgery (≥ 3 segments)	34 (72.3%)	18 (23.1%)	<0.001
Size largest CRLM (in cm)	4.2 (3.0–7.5)	3.4 (2.0–4.7)	0.001
Rectal primary tumour	15 (31.9%)	34 (43.6%)	0.195
Neoadjuvant chemotherapy	0	0	–
Adjuvant chemotherapy	0	0	–
**CLINICAL RISK SCORE**			
CRS = 3 (high score)	13 (27.7%)	10 (12.8%)	0.038
Interval CRLM < 12 months	22 (46.8%)	33 (42.3%)	0.623
CEA > 200 mg/ul	7 (16.3%)	2 (2.6%)	0.006
More than 1 CRLM	12 (25.5%)	24 (30.8%)	0.531
CRLM larger than 5 cm	17 (36.2%)	12 (15.4%)	0.008
N^+^ primary tumour	28 (59.6%)	36 (46.2%)	0.146

*CRS, clinical risk score; CEA, carcinoembryonic antigen; N^+^, lymph node positive*.

### RNA/DNA Isolation

Macrodissected frozen samples of tumour tissue and adjacent liver parenchyma were included. Both genomic DNA and total RNA were isolated from 10 μm-tissue sections (RNA/DNA purification kit, Norgen Biotek Corporation, Thorold, Ontario, Canada). DNA/RNA isolation was performed according to the manufacturer's protocol. Quality check and RNA quantification of samples was carried out by capillary electrophoresis using the LabChip GX (Perkin Elmer, Waltham, Massachusetts, USA).

### Microsatellite Instability and Mutational Hotspots

Microsatellite instability (MSI) was tested for all cases by amplifying 20 ng genomic DNA using primers for five polymorphic mononucleotide loci (*NR21, NR24, BAT25, BAT26, MONO27*). The resulting PCR products were analysed on the ABI 3730xl DNA Analyzer (Thermo Fisher, Waltham, Massachusetts, USA). The MSI status was assessed as MSI-instable when two out of five markers showed instability (18). For assessment of somatic driver mutations in *KRAS* (codon 12 and 13) and *BRAF* (V600E), genomic DNA was amplified by PCR and the resulting amplicons were analysed by Sanger sequencing. Primers are listed in Table S9.

### mRNA Sequencing and Gene Expression Quantification

Sequence libraries were generated using the Quantseq 3' mRNA sample preparation kit (Lexogen, Vienna, Austria) starting from 500 ng total RNA of each sample. Library preparation was performed according to the manufacturer's protocol. Quality check and quantification of libraries was carried out by capillary electrophoresis using the LabChip GX (Perkin Elmer). Barcoded libraries were pooled in equimolar ratios aiming at a final concentration of 2–10 pM. Sequencing was performed on an Illumina HiSeq2500, applying a 50 bp single-read protocol and aiming at 5–10 million reads per sample.

The first 12 nucleotides (nt) were trimmed of the 50nt-reads to remove sequencing artefacts. Hisat version 0.1.5-beta (19) was used to align reads to human genome reference build 37 (20). SAMtools version 1.2 (21) was used to sort the aligned reads, and gene-level quantification was then performed by HTSeq version 0.6.1p1 (22) using “–mode=union,” enabling strandedness. Because of internal poly-A priming bias, only reads mapping within 500 bp upstream from the transcript termination sites—corrected for splicing—were counted, based on Ensembl version 75 gene annotation (23). Reads in genes with multiple transcript termination sites were summed up, resulting in a read count per gene. Genes with an average read count <20 in both survival groups were excluded from further analysis.

### Data and Pathway Analysis mRNA Sequencing

Data analysis was performed in R (24). To discover possible bias, a principal component analysis was carried out on all 81 samples after read count normalization by the VSD-function of the DESeq2 package (25). Samples with <300,000 reads in the target regions were excluded from further analysis (Table S1). One sample was removed because of an undesirable patient characteristic (an additional oncological disease). Differential expression analysis using DESeq2 was performed separately for the tumour samples and adjacent liver parenchyma samples comparing the poor vs. good survivors (25). P-values were corrected for multiple testing by the Benjamini Hochberg method (False Discovery Rate, FDR). Heatmap visualizations were made after read count normalization by the regularized log transformation of the DESeq2 package, followed by mean centring per gene (25). Pathway analysis was performed by DAVID EASE software (26, 27), with p-values corrected for multiple testing by the Bonferroni method (Family Wise Error Rate, FWER).

### Immunohistochemistry

We included formalin-fixed paraffin-embedded (FFPE) samples containing both tumour and adjacent liver tissue. Besides samples from patients treated at the University Medical Centre Groningen, Groningen, the Netherlands (cohort 1), we also included samples from patients with similar inclusion criteria who were treated at the Erasmus Medical Centre, Rotterdam, the Netherlands (cohort 2). Three μm sections from FFPE tissue blocks were deparaffinised in xylene, rehydrated in graded alcohol and hematoxylin and eosin (H&E) stained. H&E stained slides were examined to confirm the inclusion of the tumour-liver transition area and to score the histopathological growth pattern (28). The Ventana automated staining system (Roche, Basel, Switzerland) was used to stain the tissue sections for CD45 (RP2/18), CD4 (SP35), CD8 (SP57), CD79A (SP18), and Kappa/Lambda (double staining, polyclonal). Staining with the primary antibodies FOXP3 (236A/E7, ABCAM, 1/100 dilution) and SLAMF7 (HPA055945, Atlas Antibodies, 1/200 dilution) was done manually. Antigen retrieval in FOXP3-stained slides was performed in a pressure cooker using a Tris/EDTA buffer (PH 9.0). Antigen retrieval in SLAMF7-stained slides was performed in the microwave using a citrate buffer (PH 6.0). Primary antibodies were diluted with 1%BSA/PBS and incubated at room temperature for 60 min. Secondary and tertiary antibodies were diluted in 1%BSA/PBS and 1% AB serum and incubated at room temperature for 30 min. Appropriate positive and negative controls were used. Diaminobenzidine (DAB) was used as the chromogen, followed by a counterstaining by hematoxylin. The staining of the immunophenotypical markers was graded by microscopic inspection in a semi-quantitative scoring system for three different areas: the invasive margin, the tumour stroma and intra-tumoural region. The invasive margin was defined as the tumour-liver transition area, the tumour stroma was defined as the area of stroma surrounding the tumour cells, and intra-tumoural staining was defined as immunopositive intra-epithelial lymphocytes in tumour cell areas. The grading of immunoreactivity was supervised by two experienced researchers and consensus was achieved in all cases. All markers were scored in grades 1–3.

### Statistical Analysis

Summary statistics were obtained using established methods and presented as percentages, median (interquartile range, IQR) or mean (standard deviation, SD). Correlation coefficients of ranked data were calculated using Spearman's rho. Variables associated with survival were first tested by univariable binary logistic regression analyses comparing poor vs. good survivors. Variables with a *p*-value <0.1 in univariable analysis were entered into the multivariable model. The used multivariable regression model was a binary logistic regression comparing poor vs. good survivors, entering all covariates simultaneously and including a constant in the model. Odds ratio (OR) and 95% confidence intervals (CI) were estimated, and a *p*-value <0.05 was considered significant. Statistical analyses were carried out with IBM SPSS Statistics V22 (IBM, Armonk, New York, USA).

## Results

### Quality Control

A summary of the mRNA sequencing quality control data is shown in Table S1. The mean (±SD) number of reads on target was 1.57 10e6 (±0.75 10e6). The variance in the dataset was explored by principal component analysis. Principal component 1 explained 78% of the total variance and showed a high concordance with tissue type (tumour vs. adjacent liver parenchyma, Figure S1). The expression of the liver-specific gene *Albumin* also resembled the differences in tissue type. Two adjacent liver samples that clustered with the tumour samples had very low levels of *Albumin* mRNA (0.10 and 0.37% of all reads in *Albumin* gene, respectively) compared to the mean *Albumin* mRNA level in the adjacent liver samples (6.21% ± 3.10) (Figure S1). Similarly, three tumour samples that clustered close to adjacent liver samples showed high levels of *Albumin* mRNA (10.69, 6.87, and 4.25%) compared to the mean *Albumin* expression in the tumour samples (0.65% ± 1.87) (Figure S1). This suggested a sampling error, and we therefore excluded these five samples from further analyses. Additionally, five samples with fewer than 300,000 reads in the target region were excluded. In total 70 samples were included for further analyses: 39 tumour samples and 31 adjacent liver samples. Of note, for 16 patients both a tumour sample and an adjacent liver sample were available. The additional 38 samples (70 minus 2^*^16) are from different patients.

### Clinicopathological Characteristics

The clinicopathological characteristics of the 39 patients of whom we included tumour samples are shown in Table S2, stratified by survival. High preoperative CEA was the only statistically different variable between both groups, with a high CEA in the poor survival group (*p* = 0.049). The clinical risk score, which is a combined score of five clinicopathological factors (5), was also significantly higher in the poor survivors (*p* = 0.031; Table S2).

The clinicopathological characteristics of the 31 patients with sampled adjacent liver parenchyma are shown in Table S3. There was no clinicopathological characteristic that was statistically different between the good and poor survivors.

### Tumour-Specific Expression Profiling

**Figure 1:**
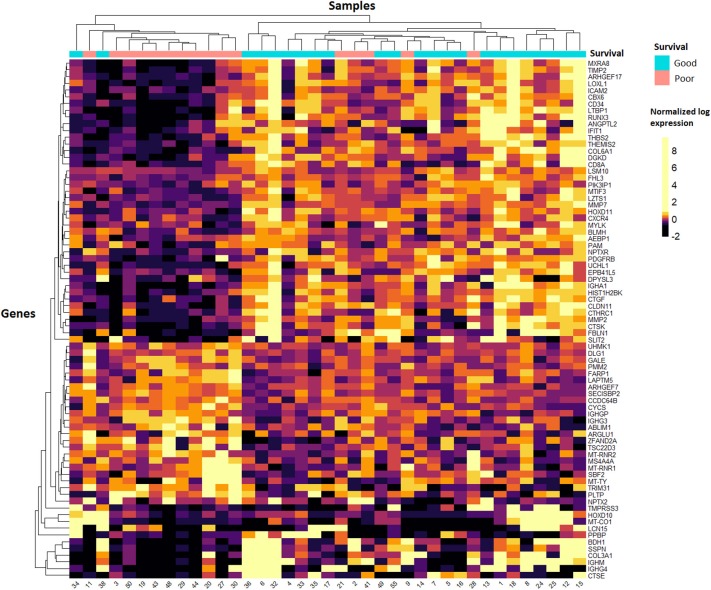
Heatmap of tumour samples. Unsupervised clustering of the 77 genes with the lowest FDR values in DESeq2 analysis. Samples are shown on the x-axis and the 77 genes on the y-axis. A quantile colour scale is used with 10 different colours ranging from black (low expression) to yellow (high expression).

After filtering for low read counts, 8,931 genes were included for further analysis by DESeq2 to identify differentially expressed genes (DEGs) between the poor vs. good survivors. Of these, 333 DEGs were differentially expressed between the two groups with a *p* <0.01, and for 77 of these DEGs the FDR was <0.1 (Table S4). Figure 1 depicts an unsupervised clustering-based heatmap of the 77 genes with a FDR <0.1, in which a clear separation of the survival groups is observed. To find out if specific biological pathways are over-represented in the 333 genes with *p* <0.01, we carried out a pathway analysis with DAVID EASE. Two biological entities appeared to be different between the survival groups (Table S5): the extracellular matrix and the immune system. The most significant GO-term-related pathway in each of these two entities was “extracellular matrix” (FWER = 1.17e-10) and “response to external stimulus” (FWER = 0.039), respectively. In general, a higher expression in both the extracellular matrix pathways and the immune-system-related pathways was observed in the good survivors.

#### Correlation With Estimate

Pathway analysis suggested that the expression of extracellular-matrix-related genes and immune-related genes is different between the survival groups (Table S5). We further analysed this using the ESTIMATE (“Estimation of STromal and Immune cells in MAlignant Tumours using Expression data”) software (29). The goal of this software package is to estimate the amount of stromal cells and immune cells by calculating a stromal score and an immune score based on 282 genes. The stromal score was higher in the good survivors (42 ± 603 vs. −469 ± 601; *p* = 0.013), as was the immune score (725 ± 660 vs. 317 ± 495; *p* = 0.043). Overall, the differences we observed in the mRNA expression data of the poor vs. good survivors are likely to be caused by the presence of immune cells and stromal cells in the tumour samples.

### Adjacent Liver Parenchyma Expression Profiling

Raw read counts of 8,772 genes were used as input for differential mRNA expression analysis by DESeq2. We compared the poor vs. good survivors to find DEGs (Table S6), and identified 109 genes with a *p* <0.01. Of these, only one gene was differentially expressed between the survival groups with a FDR <0.1: *MTRNR2L1* was more highly expressed in the good survivors (Table S6). The relatively high FDRs in this analysis indicate that the results from the adjacent liver parenchyma analysis are not as reliable as the results from the tumour samples. A pathway analysis including the 109 DEGs was performed with DAVID EASE. The transfer RNA pathway was significantly enriched and had, in general, a higher expression in the poor survivors (Table S7). Unsupervised clustering did not show uniform DEGs across the adjacent liver samples, as can be seen in the heatmap (Figure S2). The separation of the survival groups is not as clear as that seen in the tumour samples (Figure 1). Therefore, in the validation, we focused on the mRNA expression data of the tumour samples.

### Immunohistochemistry

Tissue slides of two cohorts were analysed, in which cohort 1 consisted of 47 patients (Groningen) and cohort 2 of 78 patients (Rotterdam). The samples in cohort 1 are mostly from the same patients included in the mRNA expression analysis. Table 1 shows that patients in cohort 1 had larger tumours, higher CEA levels, more frequent major liver surgery and a higher clinical risk score; Of note, all these variables are interrelated. Additionally, patients in cohort 2 had an older age.

To study the expression of stroma-related genes, a small pilot experiment was performed in which immunohistochemistry on 11 tumour tissue slides was performed using four stroma-related markers (FBLN1, MMP2, PRRX1, ABLIM1) that were differentially expressed in the mRNA expression analysis. The results did not validate the sequencing results (data not shown). We then hypothesized that the stroma-related differences might resemble the histopathological growth pattern (28). The two most prevalent growth patterns are a desmoplastic growth pattern, which is characterized by a rim of stromal cells between the tumour cells and the adjacent liver cells, and a replacement growth pattern, which lacks a desmoplastic rim. A 100% desmoplastic growth pattern was associated with good survival in both cohort 1 and 2 (*p* = 0.089 and *p* = 0.008, respectively; Table 2).

**Table 2:** Clinicopathological and biological markers vs. survival in cohorts 1, 2 and combined.

	**Cohort 1**			**Cohort 2**			**Combined cohorts**		
	**Poor survival (*n* = 21)**	**Good survival (*n* = 26)**	***P***	**Poor survival (*n* = 38)**	**Good survival (*n* = 40)**	***P***	**Poor survival (*n* = 59)**	**Good survival (*n* = 66)**	***P***
Mean follow-up in months	16.6 ± 5.5	112.3 ± 36.1	<0.001	17.0 ± 6.7	122.7 ± 36.0	<0.001	16.9 ± 6.3	118.6 ± 36.1	<0.001
**PATIENT CHARACTERISTICS**									
Mean age at liver surgery	62.6 ± 10.9	62.4 ± 8.8	0.939	71.0 ± 8.7	64.6 ± 10.1	0.004	68.0 ± 10.3	63.7 ± 9.6	0.017
Male sex	13 (61.9%)	10 (38.5%)	0.110	28 (73.3%)	22 (55.0%)	0.086	41 (69.5%)	32 (48.5%)	0.017
**TUMOUR CHARACTERISTICS**									
Major liver surgery (≥ 3 segments)	18 (85.7%)	16 (61.5%)	0.065	6 (15.8%)	12 (30%)	0.137	24 (40.7%)	28 (42.4%)	0.843
Size largest CRLM (in cm)	4.5 (3.8–12.5)	4.2 (3.0–5.2)	0.134	3.9 (2.5–4.8)	3.0 (1.8–4.5)	0.072	4.0 (2.6–5.5)	3.5 (2.0–5.0)	0.050
Rectal primary tumour	9 (42.9%)	6 (23.1%)	0.148	18 (47.4%)	16 (40%)	0.512	27 (45.8%)	22 (33.3%)	0.155
Neoadjuvant chemotherapy	0	0	–	0	0	–	0	0	–
Adjuvant chemotherapy	0	0	–	0	0	–	0	0	–
**CLINICAL RISK SCORE**									
CRS = 3 (high score)	10 (47.6%)	3 (11.5%)	0.006	5 (13.2%)	5 (12.5%)	0.931	15 (25.4%)	8 (12.1%)	0.055
Interval CRLM < 12 months	10 (47.6%)	12 (46.2%)	0.920	17 (44.7%)	16 (40%)	0.672	27 (45.8%)	28 (42.4%)	0.707
CEA > 200 mg/ul	5 (27.8%)	2 (8.0%)	0.083	1 (2.6%)	1 (2.5%)	0.971	6 (10.7%)	3 (4.6%)	0.202
More than 1 CRLM	7 (33.3%)	5 (19.2%)	0.270	15 (39.5%)	9 (22.5%)	0.104	22 (37.3%)	14 (21.2%)	0.048
CRLM larger than 5 cm	10 (47.6%)	7 (26.9%)	0.142	6 (15.8%)	6 (15%)	0.923	16 (27.1%)	13 (29.7%)	0.326
N^+^ primary tumour	14 (66.7%)	14 (53.8%)	0.373	19 (50%)	17 (42.5%)	0.507	33 (55.9%)	31 (47.0%)	0.317
**MOLECULAR CHARACTERISTICS**									
Microsatellite instability (MSI-high)	2 (9.5%)	1 (3.8%)	0.429	–	–	–	–	–	–
KRAS mutation (codon 12 and 13)	9 (42.9%)	9 (34.6%)	0.563	–	–	–	–	–	–
BRAF V600E mutation	0	0	–	–	–	–	–	–	–
**GROWTH PATTERN**									
100% desmoplastic	4 (19.0%)	11 (42.3%)	0.089	2 (5.3%)	11 (27.5%)	0.008	6 (10.2%)	22 (33.3%)	0.002
**GENERAL LYMPHOCYTES**									
High CD45 tumour stroma	3 (14.3%)	5 (19.2%)	0.654	5/37 (13.5%)	9 (22.5%)	0.307	8/58 (13.8%)	14 (21.2%)	0.281
High CD45 invasive margin	9 (42.9%)	10 (38.5%)	0.760	9 (23.7%)	9 (22.5%)	0.901	18 (30.5%)	19 (28.8%)	0.833
**T-CELLS**									
High CD4 tumour stroma	4 (19.0%)	8 (30.8%)	0.360	3/36 (8.3%)	6 (15%)	0.369	7/57 (12.3%)	14 (21.2%)	0.189
High CD4 invasive margin	7 (33.3%)	12 (46.2%)	0.373	4 (10.5%)	8 (20%)	0.246	11 (18.6%)	20 (30.3%)	0.132
High CD8 tumour stroma	12 (57.1%)	13 (50%)	0.626	17/37 (45.9%)	20 (50%)	0.722	39/58 (50%)	33 (50%)	1.000
High CD8 invasive margin	14 (66.7%)	20 (76.9%)	0.435	19 (50%)	28 (70%)	0.071	33 (55.9%)	48 (72.7%)	0.050
High CD8 intratumoural	10 (47.6%)	10 (38.5 %)	0.528	14 (36.8%)	11 (27.5%)	0.377	24 (40.7%)	21 (31.8%)	0.303
High FOXP3 tumour stroma	17 (81.0%)	24 (92.3%)	0.246	13/36 (36.1%)	25 (62.5%)	0.022	30/57 (52.6%)	49 (74.2%)	0.013
High FOXP3 invasive margin	17 (81.0%)	22 (84.6%)	0.740	24/37 (64.9%)	26 (65%)	0.990	41/58 (70.7%)	48 (72.7%)	0.801
**B-CELLS**									
High CD79A tumour stroma	9 (42.9%)	18 (69.2%)	0.069	20/37 (54.1%)	30 (75%)	0.054	29/58 (50%)	48 (72.7%)	0.009
High CD79A invasive margin	14 (66.7%)	16 (61.5%)	0.716	23 (60.5%)	33 (82.5%)	0.031	37 (62.7%)	49 (74.2%)	0.165
High K/L tumour stroma	6 (28.6%)	14 (53.8%)	0.081	11/37 (29.7%)	20 (50%)	0.070	17 (29.3%)	34 (51.5%)	0.012
High K/L invasive margin	7 (33.3%)	14 (53.8%)	0.160	16 (41.0%)	18 (45%)	0.721	23 (39.0%)	32 (48.5%)	0.285
High SLAMF7 tumour stroma	7 (33.3%)	9 (34.6%)	0.927	14/37 (37.8%)	18 (45%)	0.524	21/58 (36.2%)	27 (40.9%)	0.592
High SLAMF7 invasive margin	11 (52.4%)	15 (57.7%)	0.716	17 (44.7%)	19 (47.5%)	0.807	28 (47.5%)	34 (51.5%)	0.651

*A high CD4 and CD45 infiltration is defined as a grading of 3. In the other markers, a high infiltration was defined as a grading of ≥ 2. CRS = clinical risk score, CEA = carcinoembryonic antigen, N+ = lymph node positive*.

**Figure 2:**
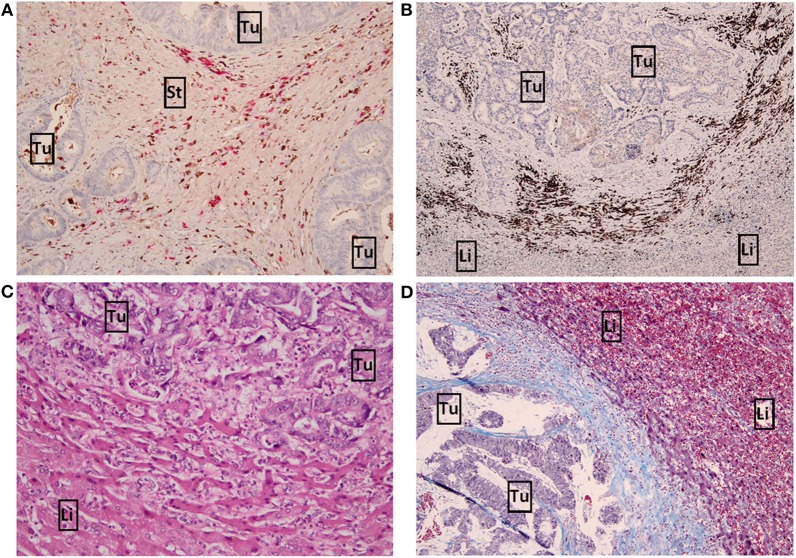
Detailed pictures of immunohistochemistry. **(A)** x10 magnification image of high Kappa/Lambda + staining in the tumour stroma. Kappa light chains are stained brown, Lambda light chains are stained pink. **(B)** x4 magnification image of high CD79A+ staining (brown) in the invasive margin. **(C)** x10 magnification image of a replacement growth pattern by H&E staining. **(D)** x10 magnification image of a desmoplastic growth pattern by Masson's trichrome staining. Tumour and liver cells are stained purple, connective tissue (desmoplastic rim) is stained blue. Tu, tumour; Li, liver; St, tumour stroma.

The mRNA expression data prompted us to stain tumour sections to validate immune-related expression, by which we chose seven immunophenotypical markers [CD45, CD4, CD8, FOXP3, CD79A, Kappa/Lambda (K/L) and SLAMF7] to study both T cell and B cell expression. Besides T cell markers we also chose to stain B cell markers as multiple immunoglobulin genes were differentially expressed between the survival groups (Table S5). CD45+ and CD4+ cells generally had a higher abundancy compared to the other markers. Figure 2 shows detailed pictures of the staining. Table 2 shows the clinicopathological characteristics and the scoring of the seven immunophenotypical markers in the poor vs. good survivors of both cohorts separately and combined. The clinical risk score was a good predictor of survival in cohort 1 (*p* =0.006; Table 2), while in cohort 2 and the combined cohort the CRS was a less good predictor of survival [*p* = 0.931 and *p* = 0.055, respectively (Table 2)]. A high CD79A expression in the tumour stroma tended to be associated with good survival in both cohort 1 and 2 (*p* = 0.069 and *p* = 0.054, respectively). Similarly, K/L expression in the tumour stroma also tended to be associated with good survival in cohorts 1 and 2 (*p* = 0.081 and *p* = 0.070, respectively). A 100% desmoplastic growth pattern tended to be associated with good survival in cohort 1 (*p* = 0.089) and was significantly associated with good survival in cohort 2 (*p* = 0.008; Table 2).

### Multivariable Analysis

A multivariable analysis was performed to test whether immunohistochemical markers have significant impact in predicting patient survival. The three most significant biomarkers were analysed in multivariable analysis (Table 3). Immunohistochemical markers that might be associated with good survival in cohorts 1 and cohort 2 were (respectively): high K/L+ infiltration in tumour stroma [*p* = 0.124; OR 2.682 (95%CI 0.762–15.248] and *p* = 0.067; OR 2.589 [95%CI 0.934–7.171)], high CD79A+ infiltration in tumour stroma [*p* = 0.110; OR 2.740 (95%CI 0.795–9.443)] and *p* = 0.095; OR 2.443 [95%CI 0.856–6.978)], and a 100% desmoplastic growth pattern [*p* = 0.157; OR 2.709 (95% CI 0.682–10.768) and *p* = 0.040; OR 5.858 (95%CI 1.083–31.693)]. These three biomarkers are significantly associated with patient survival (all *p* < 0.05) if we combine data from cohort 1 and 2 (Table 3). If we correct only for the clinicopathological factor CRS (high vs. low) in the total cohort, high K/L+ infiltration in tumour stroma [*p* = 0.026; OR 2.359 (95% CI 1.109–5.019) as high CD79A+ infiltration in tumour stroma (*p* = 0.031; OR 2.338 (95% CI 0.1.081–5.056)] as a 100% desmoplastic growth pattern [*p* = 0.007; OR 3.952 (95% CI 1.454–10.742)] were all significantly associated with favourable survival whereas the CRS was not (*p* = 0.168; *p* = 0.103; *p* = 0.163, respectively).

**Table 3:** Multivariable analysis to predict survival.

	**Univariable**		**Multivariable**	
**Factor**	***P***	**OR (95% CI)**	***P***	**OR (95% CI)**
**100% DESMOPLASTIC GROWTH PATTERN**				
Cohort 1 Cohort 2 Combined cohorts	0.096 0.017 0.003	3.117 (0.817–11.885) 6.828 (1.401–33.281) 4.417 (1.646–11.854)	0.157 0.040 0.019	2.709 (0.682–10.768) 5.858 (1.083–31.693) 3.681 (1.244–10.888)
**HIGH CD79A TUMOUR STROMA**				
Cohort 1 Cohort 2 Combined cohorts	0.086 0.057 0.010	2.917 (0.860–9.889) 2.550 (0.972–6.690) 2.667 (1.263–5.630)	0.110 0.095 0.036	2.740 (0.795–9.443) 2.443 (0.856–6.978) 2.428 (1.062–5.552)
**HIGH K/L TUMOUR STROMA**				
Cohort 1 Cohort 2 Combined cohorts	0.070 0.072 0.013	2.429 (0.930–6.341) 2.364 (0.925–6.043) 2.562 (1.218–5.389)	0.124 0.067 0.029	2.682 (0.762–15.248) 2.589 (0.934–7.171) 2.500 (1.100–5.682)

*Three biomarkers were analysed in multivariable analysis. Each biomarker is adjusted for the most significant prognostic clinical factor(s) within the respective cohort according to the “one variable per 10 events” rule (30). In cohort 1, the variables were corrected for “major liver surgery (≥ 3 segments)”. In cohort 2, correction was performed using the variables: age, sex and tumour size. In the combined cohort, correction was performed using the variables: age, sex, solitary tumour, tumour size, and cohort. An odds ratio >1 corresponds with good survival. CI, confidence interval; K/L, Kappa Lamda*.

Statistical correction for clinicopathological factors did not uniformly improve the prognostic value of biomarkers in multivariable analysis (30). This is partly explained by an association between the clinical marker tumour size and protein expression (Table S8). Of note, in patients with more than one metastasis (*n* = 36; 29% of all cases), the tumour with the largest diameter was used for immunohistochemical staining in 28 out of 36 patients. A desmoplastic growth pattern was associated with smaller tumour size [2.8 cm (1.5–4.9) vs. non-desmoplastic growth pattern 3.8 cm (2.6–5.1); *p* = 0.029]. In addition, high stromal CD79A+ infiltration was also associated with smaller tumour size [3.4 cm (2.0–4.5) vs. low CD79A+ infiltration 4.1 cm (3.0–7.0); *p* = 0.011]. Especially B cell-related markers scored in the invasive margin were associated with size of the tumour (Table S8).

## Discussion

In this study we used mRNA sequencing to identify prognostic molecular markers in patients after liver surgery for colorectal liver metastases. We selected two patient groups: poor survivors (death due to recurrence within 30 months) and good survivors (disease-free survival > 60 months after liver surgery). We show that patients with good survival had a higher expression of immune-related and stroma-related genes. Additional analysis by ESTIMATE software indicated higher immune cell and stromal cell infiltration in the tumour samples of good survivors (29). Immunohistochemistry showed in two cohorts that high immune infiltration of CD79A+ B cells and K/L+ plasma cells in tumour stroma tended to be associated with good survival. Comparable results from these biomarkers were observed in multivariable analysis correcting for clinicopathological factors. In addition, the desmoplastic growth pattern, in which a stroma-rich pseudo-capsule around the tumour is present, might also be associated with good patient survival. The prognostic value of a desmoplastic growth pattern has been reported previously (31–36). In contrast, the associations between good patient survival and high stromal infiltration of CD79A+ cells and K/L+ cells are, to the best of our knowledge, novel findings.

The top differentially expressed genes in the tumour samples in this study did not overlap with those of the four previously published expression signatures (9–12). However, no individual gene was shared between these earlier studies either (9–12). This may partly be explained by the different experimental methods used by the studies. We used mRNA sequencing, while the earlier studies used different expression microarrays (9–12, 37). The inclusion criteria for patients were also very different between studies. Our study and that of van der Stok et al. (11) did not administer neoadjuvant or adjuvant chemotherapy to included patients, while the three other studies did (9, 10, 12). Neoadjuvant chemotherapy influences tumour biology and might thereby alter gene expression levels (38). Three of the studies aimed at identifying genes that are associated with recurrence rather than with overall survival (10–12), and the cut-off values to stratify rapid recurrence vs. late or no recurrence differed per study: Snoeren et al. (10) included patients with DFS ≤ 1 year vs. DFS >1 year, Snoeren et al. (13) included patients with DFS <6 months vs. DFS > 2 years, and van der Stok et al. included patients with DFS ≤ 1 vs. DFS >3 years. In our opinion we used the most straight-forward inclusion by selecting chemotherapy-naïve patients, the strongest endpoint (disease-related overall survival; death of recurrent disease within 30 months vs. alive and free of disease 5 years after liver surgery) and the most accurate gene expression technology. These inter-study differences might explain the lack of shared genes.

We also studied adjacent liver parenchyma and found a significantly higher expression of *MTRNR2L1* in the good survivors. This gene is an isoform of *MT-RNR* (humanin), which is reported to have neuroprotective and anti-apoptotic functions and is mainly studied in age-related illness like Alzheimer's disease (39, 40).

In our study, the B-cell-related immunohistochemical markers CD79A and K/L suggest an association with patient survival. There are no previous studies that analysed CD79A and K/L in CRLM, but the prognostic value of B-cell marker CD20 was analysed by four other studies (41–44). Two studies reported an association of high CD20+ B cells in the invasive margin with a favourable overall survival (41, 44). In addition, one of these studies also showed an association between high CD20+ B cells in the tumour stroma and a favourable survival (41). Two other studies observed no associations with patient survival (42, 43). The results in literature are diverse when focussing on the role of the immune system on prognosis after surgery for CRLM (45). Although the role of tumour-infiltrating B cells and immunoglobulins is not as well-described as the role of tumour-infiltrating T cells, an association between B cell infiltration and favourable patient survival has been reported for other solid tumours (46–48). Anti-tumour immunity can be achieved through immunoglobulin production by plasma cells and, moreover, through increased cytotoxic T cell activation via antigen presenting B cells (46–48). In contrast, others have questioned the actual effect of the B cell response (47, 48). It is also proposed that the B cell infiltration is the result of IFNγ production and serves as a surrogate marker of the T-cell-mediated anti-tumour response (47).

## Conclusions

In conclusion, our study of the liver metastasis of patients with colorectal cancer showed that high stromal infiltration of CD79A+ B cells and high stromal infiltration of K/L+ plasma cells might be favourable prognostic biomarkers after surgery for CRLM. Future evaluation on external cohorts is needed to prove whether these biomarkers truly are associated with patient survival and of practical clinical value.

## Data Availability Statement

Datasets are in a publicly accessible repository: The generated and analysed RNA sequencing dataset for this study can be found in the European Nucleotide Archive (study Accession No: PRJEB35154).

## Ethics Statement

This study was approved by the institutional review board of the University Medical Center Groningen, Groningen, the Netherlands (research registry no. 201501288). Written informed consent was not required in accordance with national legislation and the institutional requirements.

## Author Contributions

JH, AG, DG, CV, RS, KJ, and KK: conceptualization and methodology. MT and KK: software. JH, LV, AG, DH, and PN: validation. JH and KK: formal analysis. JH, KK, and KJ: investigation and data curation. RS, KJ, and CV: resources. JH: writing—original draft preparation. LV, AG, DH, PN, AG, CV, RS, KJ, and KK: writing—review and editing. JH, KK, LV, and AG: visualization. LV, AG, CV, RS, KJ, and KK: supervision. KJ: project administration and funding acquisition. The samples of tumour tissue and adjacent liver parenchyma were reviewed on tissue quality by AG, experienced hepatopathologist and co-author in this study.

### Conflict of Interest

The authors declare that the research was conducted in the absence of any commercial or financial relationships that could be construed as a potential conflict of interest.
