# Supplementary material for: B Cells as Prognostic Biomarker After Surgery for Colorectal Liver Metastases
Source: Front Oncol. 2020 Mar 5;10:249. doi: 10.3389/fonc.2020.00249 (PMC7066250; doi:10.3389/fonc.2020.00249)
Supplement: Supplementary file 1 [file Data_Sheet_1.docx]

**Table S1. Quality control sequencing data**

| **Sample** | **Patient sample** | **Tissue** | **RNA quality** | **Total reads** | **Reads 500bp** | **PP Reads 500bp** | **% Alb** | **DESeq2** | **Survival** |
| --- | --- | --- | --- | --- | --- | --- | --- | --- | --- |
| 1 | 1 | T | 7.0 | 8572203 | 2415857 | 4124915 | 0.52 | y | Good |
| 2 | 1 | T | 3.9 | 7491775 | 1709058 | 4124915 | 1.09 | y | Good |
| 3 | 2 | T | 0.0 | 7180338 | 1020739 | 1658812 | 0.03 | y | Poor |
| 4 | 2 | T | 4.7 | 4287477 | 638073 | 1658812 | 0.04 | y | Poor |
| 5 | 3 | T | 4.7 | 6383603 | 1332638 | 1332638 | 0.10 | y | Poor |
| 6 | 4 | T | 4.2 | 8256476 | 2917922 | 4717742 | 0.33 | y | Good |
| 7 | 4 | T | 4.1 | 5870968 | 1799820 | 4717742 | 0.19 | y | Good |
| 8 | 5 | T | 6.4 | 6013125 | 1307188 | 1307188 | 0.13 | y | Good |
| 9 | 6 | T | 3.7 | 6875333 | 1479349 | 1479349 | 0.00 | y | Good |
| 10 | 7 | T | 6.6 | 6086419 | 2081892 | 2081892 | 0.01 | y | Good |
| 11 | 8 | T | 4.7 | 8247616 | 489752 | 1750686 | 0.02 | y | Good |
| 12 | 8 | T | 2.5 | 6417895 | 1260934 | 1750686 | 0.00 | y | Good |
| 13 | 9 | T | 5.3 | 6160323 | 1761597 | 1761597 | 0.02 | y | Poor |
| 14 | 10 | T | 4.3 | 5057575 | 192553 | 192553 | 0.05 | n | Poor |
| 15 | 11 | T | 5.0 | 7325962 | 1517751 | 1517751 | 0.01 | y | Poor |
| 16 | 12 | T | 5.4 | 15856707 | 1655381 | 2591073 | 0.00 | y | Good |
| 17 | 12 | T | 5.4 | 8518352 | 935692 | 2591073 | 0.02 | y | Good |
| 18 | 13 | T | 3.7 | 8778941 | 928313 | 1645067 | 0.00 | y | Good |
| 19 | 13 | T | 3.7 | 6484813 | 716754 | 1645067 | 0.01 | y | Good |
| 20 | 14 | T | 5.6 | 6721646 | 1524103 | 1524103 | 0.02 | y | Good |
| 21 | 15 | T | 4.5 | 8920509 | 928910 | 1502007 | 0.01 | y | Good |
| 22 | 15 | T | 4.5 | 5202267 | 573097 | 1502007 | 0.01 | y | Good |
| 23 | 16 | T | 5.5 | 7841611 | 2081843 | 2081843 | 0.01 | y | Good |
| 24 | 17 | T | 3.7 | 12803645 | 1463607 | 2324387 | 0.12 | y | Good |
| 25 | 17 | T | 3.7 | 7084015 | 860780 | 2324387 | 0.13 | y | Good |
| 26 | 18 | T | 6.3 | 7854913 | 1996631 | 1996631 | 0.00 | y | Good |
| 27 | 19 | T | 6.5 | 7810430 | 1777752 | 1777752 | 0.01 | y | Poor |
| 28 | 20 | T | 5.4 | 6638406 | 1317837 | 1317837 | 0.23 | y | Poor |
| 29 | 21 | T | 0.0 | 8057305 | 295864 | 1459644 | 1.01 | y | Poor |
| 30 | 21 | T | 5.3 | 5736561 | 1163780 | 1459644 | 1.17 | y | Poor |
| 31 | 22 | T | 4.2 | 7669517 | 2013991 | 2013991 | 6.87 | n | Good |
| 32 | 23 | T | 3.8 | 7201202 | 1883759 | 1883759 | 4.25 | n | Poor |
| 33 | 24 | T | 3.8 | 5461079 | 1031608 | 1031608 | 0.22 | y | Good |
| 34 | 25 | T | 5.7 | 6758022 | 1372277 | 1372277 | 0.00 | y | Good |
| 35 | 26 | T | 8.1 | 6753813 | 2956815 | 2956815 | 0.32 | n | Poor |
| 36 | 27 | T | 2.3 | 8663538 | 627599 | 1052004 | 0.01 | y | Poor |
| 37 | 27 | T | 3.6 | 5462207 | 424405 | 1052004 | 0.01 | y | Poor |
| 38 | 28 | T | 2.3 | 5908168 | 1039983 | 1039983 | 0.00 | y | Poor |
| 39 | 29 | T | 4.7 | 7139423 | 1781237 | 1781237 | 0.49 | y | Poor |
| 40 | 30 | T | 6.3 | 8008126 | 897925 | 1432272 | 0.00 | y | Poor |
| 41 | 30 | T | 3.5 | 4548782 | 534347 | 1432272 | 0.01 | y | Poor |
| 42 | 31 | T | 2.6 | 4485502 | 45894 | 45894 | 0.08 | n | Poor |
| 43 | 32 | T | 6.3 | 5730014 | 2174010 | 2174010 | 0.00 | y | Good |
| 44 | 33 | T | 5.0 | 5677642 | 1598180 | 1598180 | 0.00 | y | Good |
| 45 | 34 | T | 5.0 | 5626944 | 1278609 | 1278609 | 0.03 | y | Good |
| 46 | 35 | T | 6.4 | 6197356 | 2028323 | 2028323 | 0.00 | y | Good |
| 47 | 36 | T | 5.5 | 6391688 | 2334568 | 2334568 | 0.39 | y | Good |
| 48 | 37 | T | 2.5 | 4417689 | 257830 | 257830 | 0.02 | n | Good |
| 49 | 38 | T | 3.8 | 5869115 | 1003749 | 1003749 | 0.01 | y | Good |
| 50 | 39 | T | 2.9 | 4386511 | 182635 | 182635 | 0.02 | n | Poor |
| 51 | 40 | T | 3.0 | 4022107 | 118478 | 118478 | 0.09 | n | Poor |
| 52 | 41 | T | 3.6 | 7901424 | 1934589 | 1934589 | 1.49 | y | Poor |
| 54 | 43 | T | 6.3 | 6620047 | 2086405 | 2086405 | 0.01 | y | Poor |
| 55 | 44 | T | 4.2 | 7844796 | 1362774 | 1362774 | 0.04 | y | Poor |
| 59 | 48 | T | 6.8 | 7669620 | 2974072 | 2974072 | 0.17 | y | Poor |
| 60 | 49 | T | 6.3 | 7844586 | 2297708 | 2297708 | 0.12 | y | Good |
| 61 | 50 | T | 5.7 | 6966308 | 2534737 | 2534737 | 1.41 | y | Poor |
| 65 | 53 | T | 4.4 | 6738753 | 2386599 | 2386599 | 10.69 | n | Good |
| 67 | 55 | T | 5.0 | 6354199 | 2362778 | 2362778 | 1.62 | y | Good |
| 53 | 42 | L | 5.7 | 6631150 | 2594419 | 2594419 | 8.61 | y | Poor |
| 56 | 45 | L | 5.7 | 6715877 | 2535833 | 2535833 | 9.73 | y | Good |
| 57 | 46 | L | 3.4 | 6440011 | 1412489 | 1412489 | 3.05 | y | Poor |
| 58 | 47 | L | 4.1 | 7771609 | 2447460 | 2447460 | 7.09 | y | Poor |
| 62 | 51 | L | 2.6 | 5275975 | 497720 | 1615595 | 4.47 | y | Good |
| 63 | 51 | L | 3.1 | 5713298 | 1117875 | 1615595 | 7.49 | y | Good |
| 64 | 52 | L | 3.5 | 6296237 | 1277283 | 1277283 | 10.13 | y | Good |
| 66 | 54 | L | 4.5 | 6081037 | 2474934 | 2474934 | 9.67 | y | Good |
| 68 | 56 | L | 5.5 | 5047718 | 2220270 | 2220270 | 7.85 | y | Poor |
| 69 | 57 | L | 6.2 | 4800990 | 2196511 | 2196511 | 6.73 | y | Poor |
| 70 | 58 | L | NA | 4535255 | 1161536 | 1161536 | 1.06 | y | Poor |
| 71 | 59 | L | 6.6 | 5553831 | 2632808 | 2632808 | 7.64 | y | Poor |
| 72 | 60 | L | 4.8 | 4906508 | 1307219 | 1307219 | 0.10 | n | Poor |
| 73 | 61 | L | 4.4 | 4681506 | 1865049 | 1865049 | 13.82 | y | Good |
| 74 | 62 | L | 6.1 | 5173344 | 2214967 | 2214967 | 0.37 | n | Poor |
| 75 | 63 | L | 3.5 | 4720130 | 1858090 | 1858090 | 6.56 | y | Poor |
| 76 | 64 | L | 7.2 | 4874999 | 2446310 | 2446310 | 8.46 | y | Poor |
| 77 | 65 | L | 6.4 | 4718837 | 2001195 | 2001195 | 6.19 | y | Good |
| 78 | 66 | L | 4.0 | 4111092 | 1554401 | 1554401 | 10.33 | y | Poor |
| 79 | 67 | L | 5.1 | 5298327 | 1980435 | 1980435 | 3.74 | y | Good |
| 80 | 68 | L | 2.9 | 1821079 | 351371 | 1198692 | 3.96 | y | Good |
| 81 | 68 | L | 2.9 | 4654880 | 847321 | 1198692 | 3.73 | y | Good |
| 82 | 69 | L | 8.5 | 5611426 | 2980695 | 2980695 | 4.67 | y | Poor |
| 83 | 70 | L | 6.3 | 5117309 | 2540612 | 2540612 | 7.53 | y | Good |
| 84 | 71 | L | 6.7 | 4961243 | 2579805 | 2579805 | 7.91 | y | Poor |
| 85 | 72 | L | 5.6 | 5770237 | 2663802 | 2663802 | 8.17 | y | Good |
| 86 | 73 | L | 1.4 | 3666318 | 941809 | 941809 | 1.94 | y | Poor |
| 87 | 74 | L | 4.7 | 4717996 | 1982690 | 1982690 | 5.61 | y | Good |
| 88 | 75 | L | 5.5 | 4596363 | 2046311 | 2046311 | 8.41 | y | Poor |
| 89 | 76 | L | 6.7 | 4575768 | 2007289 | 2007289 | 6.32 | y | Good |
| 90 | 77 | L | 6.1 | 4290394 | 1222294 | 1222294 | 3.36 | y | Poor |
| 91 | 78 | L | 4.5 | 5002237 | 2087948 | 2087948 | 7.33 | y | Poor |
| 92 | 79 | L | NA | 3124886 | 945813 | 945813 | 1.99 | y | Good |
| 93 | 80 | L | 4.1 | 4443572 | 1377349 | 1377349 | 6.08 | y | Poor |
| 94 | 81 | L | 5.5 | 3931154 | 1287460 | 1287460 | 4.04 | y | Good |

In some cases, two samples per patient were analysed. The RNA quality score has a range of 0-10. Tissue: ‘T’ = tumour sample, ‘L’ = adjacent liver sample. Total reads = number total generated reads before aligning to the reference genome. Reads 500bp = number of reads in the target region. PP reads 500bp = number of reads in the target region, calculated per patient. % Alb = percentage of reads that aligned to the *Albumin* gene. DESeq2 shows which samples are used for differential expression analysis (y = yes, n = no).

|  | **Poor survival**  **(<30 months, n=16)** | **Good survival**  **(>60 months, n=23)** | **P value** |
| --- | --- | --- | --- |
| Mean follow-up in months | 17.0 ± 5.2 | 108.6 ± 35.2 | <0.001 |
|  |  |  |  |
| **Patient characteristics** |  |  |  |
| Mean age at liver surgery | 62.8 ± 10.5 | 63.7 ± 7.4 | 0.814 |
| Male sex | 9 (56.3%) | 8 (34.8%) | 0.184 |
|  |  |  |  |
| **Tumour characteristics** |  |  |  |
| Major liver surgery (≥ 3 segments) | 13 (81.3%) | 15 (65.2%) | 0.274 |
| Size largest CRLM (cm) | 7.5 ± 5.2 | 4.6 ± 2.6 | 0.056 |
| Rectal primary tumour | 8 (50%) | 6 (26.1%) | 0.126 |
| Neoadjuvant chemotherapy | 0 | 0 | - |
|  |  |  |  |
| **Molecular characteristics** |  |  |  |
| Microsatellite instability (MSI-high) | 1 (6.3%) | 1 (4.3%) | 0.791 |
| KRAS mutation (codon 12 and 13) | 6 (37.5%) | 8 (34.8%) | 0.862 |
| BRAF V600E mutation | 0 | 0 | - |
|  |  |  |  |
| **Clinical risk score** |  |  |  |
| CRS = 3 (high score) | 7 (43.8%) | 3 (13.0%) | 0.031 |
| Interval CRLM <12 months | 7 (43.8%) | 10 (43.5%) | 0.987 |
| CEA >200 mg/ul | 5 (31.3%) | 2 (8.7%) | 0.049 |
| More than 1 CRLM | 3 (18.8%) | 5 (21.7%) | 0.820 |
| CRLM larger than 5cm | 7 (43.8%) | 9 (39.1%) | 0.773 |
| N+ primary tumour | 12 (75%) | 11 (47.8%) | 0.090 |

**Table S2. Clinicopathological characteristics tumour samples**

CRS = clinical risk score, CEA = carcinoembryonic antigen, N+ = lymph node positive

**Table S3. Clinicopathological characteristics adjacent liver samples**

CRS = clinical risk score, CEA = carcinoembryonic antigen, N+ = lymph node positive

|  | **Poor survival**  **(<30 months, n=17)** | **Good survival**  **(>60 months, n=14)** | **P value** |
| --- | --- | --- | --- |
| Mean follow-up in months | 17.6 ± 4.8 | 118.9 ± 41.4 | <0.001 |
|  |  |  |  |
| **Patient characteristics** |  |  |  |
| Mean age at liver surgery | 66.8 ± 10.5 | 63.2 ± 7.4 | 0.298 |
| Male sex | 8 (47.1%) | 9 (64.3%) | 0.337 |
|  |  |  |  |
| **Tumour characteristics** |  |  |  |
| Major liver surgery (≥ 3 segments) | 10 (58.8%) | 10 (71.4%) | 0.465 |
| Size largest CRLM (in cm) | 5.61 ± 3.29 | 5.16 ± 2.88 | 0.750 |
| Rectal primary tumour | 6 (35.3%) | 2 (14.3%) | 0.183 |
| Neoadjuvant chemotherapy | 0 | 0 | - |
|  |  |  |  |
| **Clinical risk score** |  |  |  |
| CRS = 3 (high score) | 7 (41.2%) | 2 (14.3%) | 0.101 |
| Interval CRLM <12 months | 8 (47.1%) | 7 (50%) | 0.870 |
| CEA >200 mg/μl | 2 (11.8%) | 1 (7.1%) | 0.626 |
| More than 1 CRLM | 6 (35.3%) | 3 (21.4%) | 0.397 |
| CRLM larger than 5cm | 9 (52.9%) | 8 (57.1%) | 0.815 |
| N+ primary tumour | 13 (76.5%) | 7 (50%) | 0.125 |

**Table S4. Tumour DESeq2 analysis**

| **Ensembl code** | **Gene symbol** | **Log2 fold change** | **P-value** | **FDR** |
| --- | --- | --- | --- | --- |
| ENSG00000210082 | *MT-RNR2* | 1.03 | 8.04E-07 | 0.006392 |
| ENSG00000211459 | *MT-RNR1* | 1.27 | 1.43E-06 | 0.006392 |
| ENSG00000137673 | *MMP7* | -1.97 | 6.17E-06 | 0.009369 |
| ENSG00000154277 | *UCHL1* | -1.43 | 6.08E-06 | 0.009369 |
| ENSG00000145147 | *SLIT2* | -1.16 | 5.26E-06 | 0.009369 |
| ENSG00000102606 | *ARHGEF7* | 0.67 | 6.29E-06 | 0.009369 |
| ENSG00000181817 | *LSM10* | -0.63 | 8.37E-06 | 0.010684 |
| ENSG00000128710 | *HOXD10* | -2.91 | 1.14E-05 | 0.012747 |
| ENSG00000211892 | *IGHG4* | -3.15 | 2.54E-05 | 0.023714 |
| ENSG00000178381 | *ZFAND2A* | 1.34 | 2.66E-05 | 0.023714 |
| ENSG00000106236 | *NPTX2* | -3.31 | 3.73E-05 | 0.023766 |
| ENSG00000211895 | *IGHA1* | -2.53 | 3.63E-05 | 0.023766 |
| ENSG00000168542 | *COL3A1* | -1.22 | 3.22E-05 | 0.023766 |
| ENSG00000160183 | *TMPRSS3* | 1.83 | 2.97E-05 | 0.023766 |
| ENSG00000211897 | *IGHG3* | -2.35 | 5.45E-05 | 0.028641 |
| ENSG00000108578 | *BLMH* | -0.73 | 5.27E-05 | 0.028641 |
| ENSG00000161267 | *BDH1* | 0.93 | 5.22E-05 | 0.028641 |
| ENSG00000204616 | *TRIM31* | 1.39 | 7.35E-05 | 0.034656 |
| ENSG00000210144 | *MT-TY* | 1.67 | 7.37E-05 | 0.034656 |
| ENSG00000100100 | *PIK3IP1* | -0.67 | 8.21E-05 | 0.036660 |
| ENSG00000163736 | *PPBP* | -2.90 | 9.62E-05 | 0.038794 |
| ENSG00000100979 | *PLTP* | -1.12 | 0.000108 | 0.038794 |
| ENSG00000113657 | *DPYSL3* | -1.06 | 0.000130 | 0.038794 |
| ENSG00000065534 | *MYLK* | -1.03 | 0.000109 | 0.038794 |
| ENSG00000110237 | *ARHGEF17* | -0.95 | 0.000127 | 0.038794 |
| ENSG00000157514 | *TSC22D3* | -0.89 | 0.000108 | 0.038794 |
| ENSG00000140650 | *PMM2* | 0.56 | 0.000130 | 0.038794 |
| ENSG00000122033 | *MTIF3* | 0.65 | 0.000125 | 0.038794 |
| ENSG00000197903 | *HIST1H2BK* | 0.99 | 0.000105 | 0.038794 |
| ENSG00000172115 | *CYCS* | 1.13 | 0.000120 | 0.038794 |
| ENSG00000099204 | *ABLIM1* | 0.88 | 0.000141 | 0.040518 |
| ENSG00000162576 | *MXRA8* | -1.28 | 0.000149 | 0.041586 |
| ENSG00000211899 | *IGHM* | -2.16 | 0.000182 | 0.049160 |

Genes with FDR < 0.05 are presented, ranked from low-high. Genes with higher expression in poor survivors have a positive log2 fold change, genes with higher expression in good survivors have a negative log2 fold change. p-value is corrected by the Benjamini-Hochberg method (FDR, right column).

**Table S5. Pathway analysis of the tumour samples by DAVID EASE**

| **Biological pathway** | **EASE score** | **FWER** | **Genes in biological pathway** |
| --- | --- | --- | --- |
| Extracellular matrix | 1.26E-13 | 1.17E-10 | *ADAMTS2, ASPN, COL10A1, COL11A1, COL15A1, COL3A1, COL5A1, COL6A1, COL6A3, COL8A1, COMP, CTGF, FBLN1, FBLN5, FMOD, ITGBL1, LAMA4, LTBP2, LUM, MGP, MMP14, MMP2, MMP7, SPARC, SSPN, THBS2, TIMP2, TIMP3, VAT1* |
| Cell adhesion | 5.44E-07 | 5.07E-04 | *AEBP1, ARHGDIB, CCL5, CD34, CLDN11, COL11A1, COL15A1, COL5A1, COL6A1, COL6A3, COL8A1, COMP, CTGF, EDIL3, FBLN5, GNE, ICAM2, ITGBL1, LAMA4, MCAM, NPTX2, PKP3, SELPLG, SLIT2, SSPN, THBS2, TSTA3* |
| Collagen | 2.62E-06 | 2.44E-03 | *COL10A1, COL11A1, COL15A1, COL3A1, COL5A1, COL6A1, COL6A3, COL8A1* |
| Response to external stimulus | 4.18E-05 | 3.90E-02 | *ABLIM1, ARHGDIB, B2M, CCL5, CD3G, CD53, CD8A, COL11A1, CTGF, CXCR4, CYP1B1, CYP3A4, DEFA6, DGKD, F2R, GPX1, HOMER3, IFIT1, IFIT3, IGHA1, IGHG1, IGHG3, IGHG4, IGHM, LGALS3BP, LTB, LUM, MGLL, MMP14, PHYH, PIK3CB, PMP22, PON1, PPBP, RNASE6, SH2D2A, SLIT2, SPINK5, TIMP3, TMPRSS3, TNFSF13B, TRBC2* |
| Receptor binding | 3.14E-04 | 2.93E-01 | *ANGPTL2, CCL5, CD3G, CD8A, CTGF, DKK3, EDIL3, F2R, FBLN5, ICAM2, LTB, PDGFC, PPBP, S100A10, SELPLG, SLIT2, TNFSF13B, WNT5A* |
| Antigen binding | 1.90E-03 | 1.00 | *IGHA1, IGHG1, IGHG3, IGHG4, IGH* |
| Defence response | 1.93E-03 | 1.00 | *ARHGDIB, B2M, CCL5, CD3G, CD53, CD8A, CXCR4, DEFA6, DGKD, HOMER3, IFIT1, IFIT3, IGHA1, IGHG1, IGHG3, IGHG4, IGHM, LGALS3BP, LTB, MGLL, PPBP, RNASE6, SH2D2A, SPINK5, TNFSF13B, TRBC2* |

Pathways listed are a selection of the enriched pathways based on the 333 genes with a FDR < 0.1. EASE score = upper bound of the distribution of Jackknife Fisher exact probabilities given the enriched genes compared to the reference genes. FWER = EASE score adjusted for multiple testing by the Bonferroni method.

**Table S6. Adjacent liver samples DESeq2 analysis**

| **Ensembl code** | **Gene symbol** | **Log2 fold change** | **P-value** | **FDR** |
| --- | --- | --- | --- | --- |
| ENSG00000256618 | *MTRNR2L1* | -3.66 | 4.64E-08 | 0.000407 |
| ENSG00000117877 | *CD3EAP* | -1.49 | 5.23E-05 | 0.221632 |
| ENSG00000101413 | *RPRD1B* | -1.56 | 7.58E-05 | 0.221632 |
| ENSG00000185404 | *SP140L* | -1.06 | 0.000409 | 0.650083 |
| ENSG00000116990 | *MYCL* | 1.21 | 0.000450 | 0.650083 |
| ENSG00000115602 | *IL1RL1* | -1.64 | 0.000512 | 0.650083 |
| ENSG00000172671 | *ZFAND4* | -0.78 | 0.000608 | 0.650083 |
| ENSG00000227057 | *WDR46* | -0.72 | 0.000728 | 0.650083 |
| ENSG00000163638 | *ADAMTS9* | -1.30 | 0.000740 | 0.650083 |
| ENSG00000251259 | *AC004069.2* | -0.89 | 0.000814 | 0.650083 |
| ENSG00000158825 | *CDA* | 0.91 | 0.000977 | 0.650083 |
| ENSG00000122547 | *EEPD1* | -0.82 | 0.001005 | 0.650083 |
| ENSG00000055147 | *FAM114A2* | -0.44 | 0.001111 | 0.650083 |
| ENSG00000178966 | *RMI1* | 0.58 | 0.001306 | 0.650083 |
| ENSG00000174738 | *NR1D2* | 0.73 | 0.001350 | 0.650083 |
| ENSG00000163938 | *GNL3* | -0.90 | 0.001438 | 0.650083 |
| ENSG00000151914 | *DST* | -1.04 | 0.001507 | 0.650083 |
| ENSG00000221914 | *PPP2R2A* | -0.47 | 0.001642 | 0.650083 |
| ENSG00000164144 | *ARFIP1* | -0.39 | 0.001864 | 0.650083 |
| ENSG00000163811 | *WDR43* | -0.85 | 0.001877 | 0.650083 |
| ENSG00000115942 | *ORC2* | -0.69 | 0.002005 | 0.650083 |
| ENSG00000138166 | *DUSP5* | -0.98 | 0.002054 | 0.650083 |
| ENSG00000245532 | *NEAT1* | -1.55 | 0.002054 | 0.650083 |
| ENSG00000020577 | *SAMD4A* | -0.65 | 0.002191 | 0.650083 |
| ENSG00000062716 | *VMP1* | -0.73 | 0.002228 | 0.650083 |
| ENSG00000198650 | *TAT* | 1.21 | 0.002232 | 0.650083 |
| ENSG00000105792 | *C7orf63* | -0.82 | 0.002296 | 0.650083 |
| ENSG00000106105 | *GARS* | -0.77 | 0.002399 | 0.650083 |
| ENSG00000170448 | *NFXL1* | -0.89 | 0.002470 | 0.650083 |
| ENSG00000173227 | *SYT12* | -1.30 | 0.002499 | 0.650083 |

Genes with p-value < 0.0025 are presented in this table, ranked from low to high. Genes with a higher expression in the poor survivors have a positive log2 fold change. Genes with a higher expression in the good survivors have a negative log2 fold change. The p-value is corrected by the Benjamini-Hochberg method (FDR, right column).

**Table S7. Pathway analysis of the adjacent liver samples by DAVID EASE**

| **Biological pathway** | **EASE score** | **FWER** | **Genes in biological pathway** |
| --- | --- | --- | --- |
| tRNA metabolism | 1.16E-05 | 5.36E-03 | *CCT2, GARS, SYT12, TARS, WARS, WDR4, YARS* |
| tRNA ligase activity | 9.38E-04 | 4.35E-01 | *CCT2, GARS, TARS, WARS, YARS* |
| Translation | 4.41E-03 | 1.00 | *CCT2, GARS, SYT12, TARS, WARS, YARS* |
| Binding | 1.19E-02 | 1.00 | *ADAMTS13, ADAMTS9, BDP1, CCL19, CCT2, CDA, CLK1, CRELD1, ELL, FBXO32, FNBP4, GARS, HIF1A, IGHG4, IL1RL1, IL4R, ILF3, KIAA0020, LIMK2, MX2, MYBBP1A, NFXL1, NR1D2, PPP2R2A, S100A9, SOCS2, SSBP4, STK17A, TARS, TAT, TCERG1, TGFBR1, THOC1, TNFSF14, VCL, WARS, WDR4, YAP1, YARS, ZNF274* |

Pathways presented are a selection of the enriched pathways based on the 109 genes with p-value < 0.01. EASE score = Upper bound of the distribution of Jackknife Fisher exact probabilities given the enriched genes compared to the reference genes. FWER = EASE score adjusted for multiple testing correction by the Bonferroni method.

**Table S8. Immunohistochemical markers vs. diameter of stained CRLM**

|  | **Diameter CRLM (in cm) in marker with low expression** | **Diameter CRLM (in cm) in marker with high expression** | **P-value** |
| --- | --- | --- | --- |
| **General lymphocytes** |  |  |  |
| CD45 tumour stroma | 3.5 (2.0-5.0) | 3.9 (2.5-5.1) | 0.598 |
| CD45 invasive margin | 3.6 (2.1-5.1) | 3.2 (2.4-4.3) | 0.285 |
|  |  |  |  |
| **T-cells** |  |  |  |
| CD4 tumour stroma | 3.5 (2.0-5.0) | 4.0 (2.8-5.0) | 0.657 |
| CD4 invasive margin | 3.5 (2.0-5.0) | 3.7 (2.5-4.5) | 0.904 |
| CD8 tumour stroma | 3.6 (2.4-4.7) | 3.5 (2.0-5.5) | 0.799 |
| CD8 invasive margin | 4.5 (3.0-6.4) | 3.2 (2.0-4.5) | 0.003 |
| CD8 intratumoural | 4.0 (3.0-5.1) | 2.5 (1.5-4.5) | 0.002 |
| FOXP3 tumour stroma | 3.5 (2.1-5.0) | 3.5 (2.5-5.0) | 0.933 |
| FOXP3 invasive margin | 4.5 (3.0-6.0) | 3.4 (2.0-4.5) | 0.017 |
|  |  |  |  |
| **B-cells** |  |  |  |
| CD79A tumour stroma | 4.1 (3.0-7.0) | 3.4 (2.0-4.5) | 0.011 |
| CD79A invasive margin | 4.7 (3.4-8.5) | 3.1 (2.0-4.5) | <0.001 |
| K/L tumour stroma | 3.8 (2.4-5.5) | 3.5 (2.0-4.5) | 0.221 |
| K/L invasive margin | 4.0 (2.8-5.5) | 3.2 (2.0-4.5) | 0.077 |
| SLAMF7 tumour stroma | 3.8 (2.6-5.0) | 3.1 (2.0-4.9) | 0.170 |
| SLAMF7 invasive margin | 4.1 (3.0-5.5) | 3.0 (2.0-4.5) | 0.002 |

A high CD4 and CD45 infiltration is defined as a grading of 3. In the other markers, high infiltration was defined as a grading of ≥ 2. K/L = Kappa/Lambda

|  | **Forward primer** | **Reverse primer** |
| --- | --- | --- |
| **Microsatellite** |  |  |
| NR21 | TAAATGTATGTCTCCCCTGG | ATTCCTACTCCGCATTCACA |
| NR24 | CCATTGCTGAATTTTACCTC | ATTGTGCCATTGCATTCCAA |
| MONO27 | CACTCCAGCGTGGGAGACAG | GGTGGATCAAATTTCACTTGG |
| BAT25 | TCGCCTCCAAGAATGTAAGT | TCTGCATTTTAACTATGGCTC |
| BAT26 | TGACTACTTTTGACTTCAGCC | TAACCATTCAACATTTTTAACCC |
|  |  |  |
| **Mutation hotspot** |  |  |
| KRAS codon 12 and 13 | CGATACACGTCTGCAGTCAA | GAATGGTCCTGCACCAGTAA |
| BRAF V600E | ATAATGCTTGCTCTGATAGG | TGTGAATACTGGGAACTATG |

**Table S9. Primer specifics**


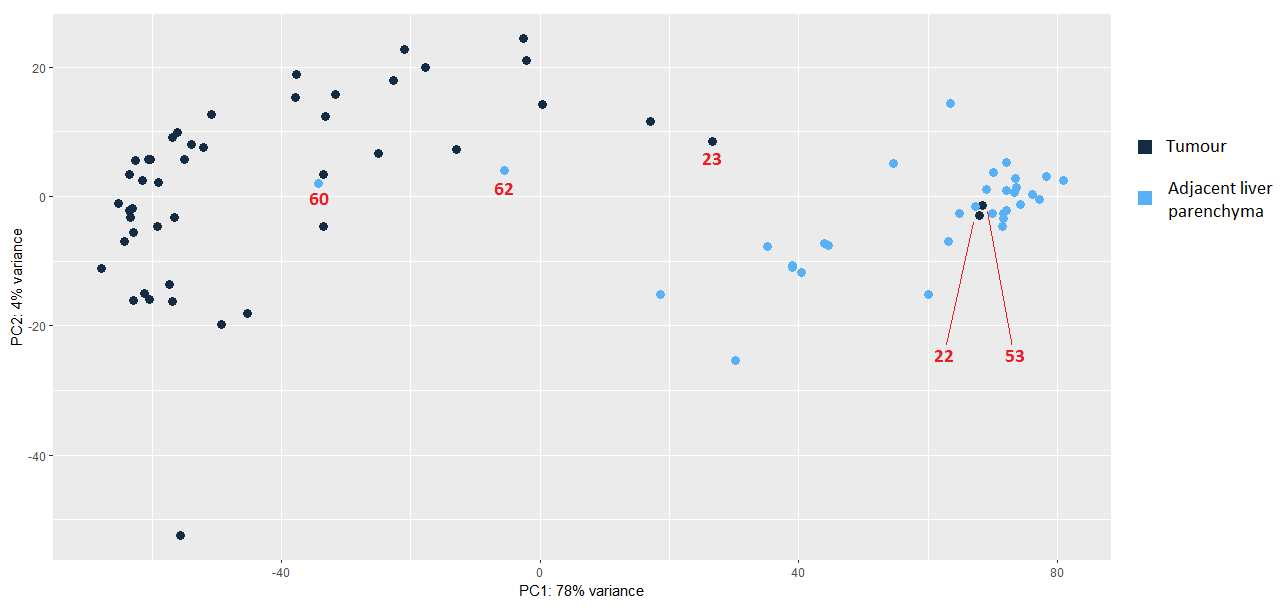


**Figure S1. Principal component analysis of all 81 patient samples**

Principal component 1 (PC1) explains 78% of the variance in the dataset and is plotted on the x-axis. Principal component 2 (PC2) explains 4% of the variance in the dataset and is plotted on the y-axis. Tumour samples are coloured dark blue and adjacent liver parenchyma samples are coloured light blue. Samples 60,62,23,22 and 53 were removed from downstream analyses based on this plot and the percentage of reads in the *Albumin* gene. Adjacent liver parenchyma samples 60 and 62 had low *Albumin* reads (0.1% and 0.4%, respectively) and tumour samples 23,22 and 53 had high *Albumin* reads (4.3%, 6.9% and 10.7%, respectively).


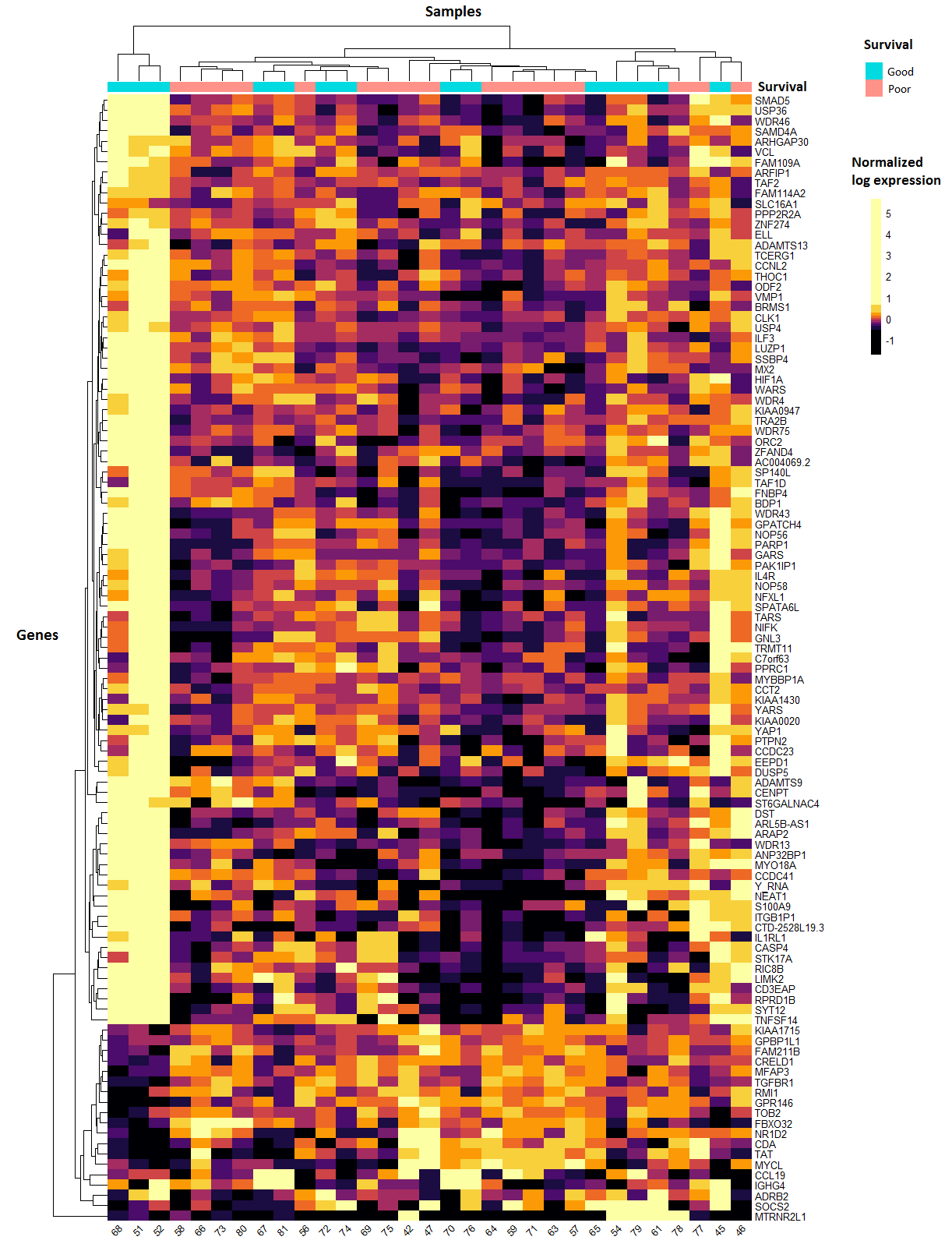


**Figure S2. Heatmap adjacent liver parenchyma samples**

Unsupervised clustering of the 109 genes with the lowest FDR values in DESeq2 analysis. Samples are shown on the x-axis and the 109 genes on the y-axis. A quantile colour scale is used with 10 different colours ranging from black (low expression) to yellow (high expression).
